# Influence of Reduced Folate Carrier and Aminoimidazole Carboxamide Ribonucleotide Transformylase gene polymorphisms on the efficacy of methotrexate in juvenile idiopathic arthritis

**DOI:** 10.1186/1546-0096-9-S1-P120

**Published:** 2011-09-14

**Authors:** Yelda Bilginer, Eda Utine, Özgür Kasapçopur, Bora Gülhan, Erkan Demirkaya, Rezan Topaloğlu, Ali Düzova, Fatih Özaltın, Mehmet Alikaşifoğlu, Seza Özen

**Affiliations:** 1 Hacettepe University Faculty of Medicine Pediatric Rheumatology and Nephrology Unit, Turkey; 2 Hacettepe University Faculty of Medicine Department of Pediatrics Clinical Genetics Unit, Turkey; 3 İstanbul University Cerrahpaşa Medical School Department of Pediatric Rheumatology, Turkey

## Background

Methotrexate (MTX) is one of the most common disease modifying antirheumatic drugs used for the treatment of juvenile idiopathic arthritis (JIA). Reliable predictors for the efficacy of MTX therapy would be very helpful for the development of more personalized therapy in an early stage of the disease.

## Aim

We investigated whether Reduced Folate Carrier (RFC) (C347G), and Aminoimidazole Carboxamide Ribonucleotide Transformylase (ATIC) (G80A) gene polymorphisms were related to the efficacy of methotrexate in JIA patients with a polyarticular course.

## Methods

Ninety seven JIA patients with a polyarticular course were enrolled into the study. All patients received methotrexate treatment for at least for 6 months. The efficacy of treatment was judged by improvement in physician’s global assesment, the number of affected joints, erythrocyte sedimentation rate and C-reactive protein. RFC and ATIC gene polymorphisms were determined by polymerase chain reaction- restriction fragment length polymorphism ( PCR-RFLP) analysis.

## Results

The median age of the patients was 72 (12-92) months. The response rate to MTX was 45%. There was no significant association between RFC and ATIC polymorphisms and efficacy of MTX treatment (p: 0.254, p: 0.098 respectively).

## Conclusion

In this study the response to MTX was not associated with RFC and ATIC polymorphisms. The pharmacogenomics related to MTX is probably quite complex. One probably needs to study all polymorphisms in the related enzymes and associated pathways to predict response to MTX.

